# Targeting inhibitory phagocytosis checkpoints in cancer immunotherapy: mechanisms, therapeutic strategies, and translational challenges

**DOI:** 10.3389/fimmu.2026.1924227

**Published:** 2026-10-13

**Authors:** Jie Li, Mei Ding, Jian Sun, Lu Gao, Zhencang Zhou, Qian Zeng, Fang Xu, Siming Li, Zuochen Du, Pei Huang, Yan Chen

**Affiliations:** 1 Department of Pediatrics, Affiliated Hospital of Zunyi Medical University, Zunyi, Guizhou, China; 2 Guizhou Children’s Hospital, Zunyi, Guizhou, China; 3 Guizhou Biomanufacturing Laboratory, Affiliated Hospital of Zunyi Medical University, Zunyi, Guizhou, China

**Keywords:** “Don’t Eat Me” signals, cancer immunotherapy, CD47–SIRPα axis, glyco-immune barrier, inhibitory phagocytosis checkpoints, macrophages

## Abstract

Tumors evade macrophage-mediated clearance through mechanistically distinct but functionally convergent anti-phagocytic pathways. This review examines canonical and emerging receptor–ligand “don’t eat me” axes, Siglec-dependent glyco-inhibitory checkpoints, and an emerging noncanonical glycocalyx barrier associated with sialylated CD43, linking these mechanisms to impaired macrophage recognition and activation, phagocytic synapse formation, and target-cell engulfment. We discuss their molecular basis, roles in tumor immune evasion, and therapeutic targeting, with emphasis on checkpoint-blocking antibodies, Fc-engineered antibodies, bispecific molecules, fusion proteins, and glyco-immune interventions. Clinical translation has nevertheless been uneven because of on-target/off-tumor toxicity, insufficient tumor selectivity, insufficient pro-phagocytic signaling, macrophage heterogeneity, and parallel immunosuppressive pathways. The strength and maturity of the supporting evidence vary considerably across targets, with CD47–SIRPα remaining the most extensively studied and clinically developed axis. Further progress will require mechanism- and evidence-guided target prioritization, biomarker-informed patient selection, and drug engineering to improve therapeutic selectivity and therapeutic windows; the clinical value of multi-checkpoint and other combination strategies remains to be established.

## 1 Introduction

Macrophage-mediated phagocytosis of intact cells is a key component of innate tumor immunosurveillance and is governed by the dynamic balance between pro-phagocytic signals and inhibitory signals that preserve self-tolerance and restrain inappropriate cell clearance (1, 2). Tumor cells can exploit this balance by sustaining or enhancing anti-phagocytic signals, thereby impairing target-cell recognition, phagocytic synapse formation, and cytoskeletal remodeling to evade macrophage-mediated clearance (1, 3, 4). The CD47–SIRPα axis was the first inhibitory phagocytosis checkpoint to be established and remains the most extensively studied (5). Blockade of this axis has shown antitumor activity in preclinical and early-phase clinical studies (6–8); however, the broad expression of CD47 in normal tissues, associated toxicity, and heterogeneous clinical efficacy (9–11) underscore the translational challenges of targeting phagocytosis checkpoints.

The spectrum of tumor anti-phagocytic mechanisms has expanded beyond the CD47–SIRPα axis to include multiple receptor–ligand “don’t eat me” signals (5), Siglec-dependent glyco-inhibitory checkpoints (12), and a noncanonical CD43-mediated glycocalyx barrier (13). These mechanisms differ in molecular architecture, mode of action, and maturity of the supporting evidence, yet converge functionally in limiting macrophage-mediated tumor cell clearance.

This review compares the structural and physiological basis of inhibitory phagocytosis checkpoints, their functional hijacking by tumors, therapeutic approaches, and evidence for clinical translation. We further examine key factors that determine tumor selectivity and therapeutic windows and critically assess the mechanistic rationale and evidentiary limitations of combination strategies.

## 2 Inhibitory phagocytosis checkpoints

In 2000, the CD47–SIRPα axis was established as a prototypical inhibitory phagocytosis checkpoint, providing the first clear mechanistic link between “don’t eat me” signaling and suppression of macrophage-mediated phagocytosis (5). The conceptual landscape of tumor anti-phagocytic mechanisms has since expanded from this single axis to a multilayered inhibitory system (**Figure 1**). Canonical receptor–ligand inhibitory phagocytosis checkpoints include CD47–SIRPα, MHC-I–LILRB1, PD-1–PD-L1, CD200–CD200R1, and SLAMF3/SLAMF4, which primarily restrain macrophage activation and tumor cell phagocytosis through specific ligand–receptor interactions and downstream inhibitory signaling (14–16). Siglec-dependent glyco-inhibitory checkpoints, including CD24–Siglec-10, α3β1 integrin–Siglec-10, and GD2–Siglec-7, rely on recognition of tumor-cell surface glycans or glycolipids by inhibitory Siglec receptors, thereby converting tumor-associated glycan or glycolipid features into immunosuppressive inputs that limit macrophage-mediated tumor clearance (12, 17). In parallel, highly O-glycosylated and sialylated CD43 can form a dense glycocalyx on tumor cells that sterically restricts cell–cell contact and phagocytic synapse formation, producing a noncanonical anti-phagocytic effect (13). Collectively, these findings show that tumor-mediated resistance to phagocytosis extends beyond canonical receptor–ligand checkpoints to Siglec-dependent glyco-inhibitory checkpoints and glycocalyx-mediated barriers, broadening the field from a CD47–SIRPα-centric model toward a more integrated framework of anti-phagocytic mechanisms (**Figure 2**).

**Figure 1:**
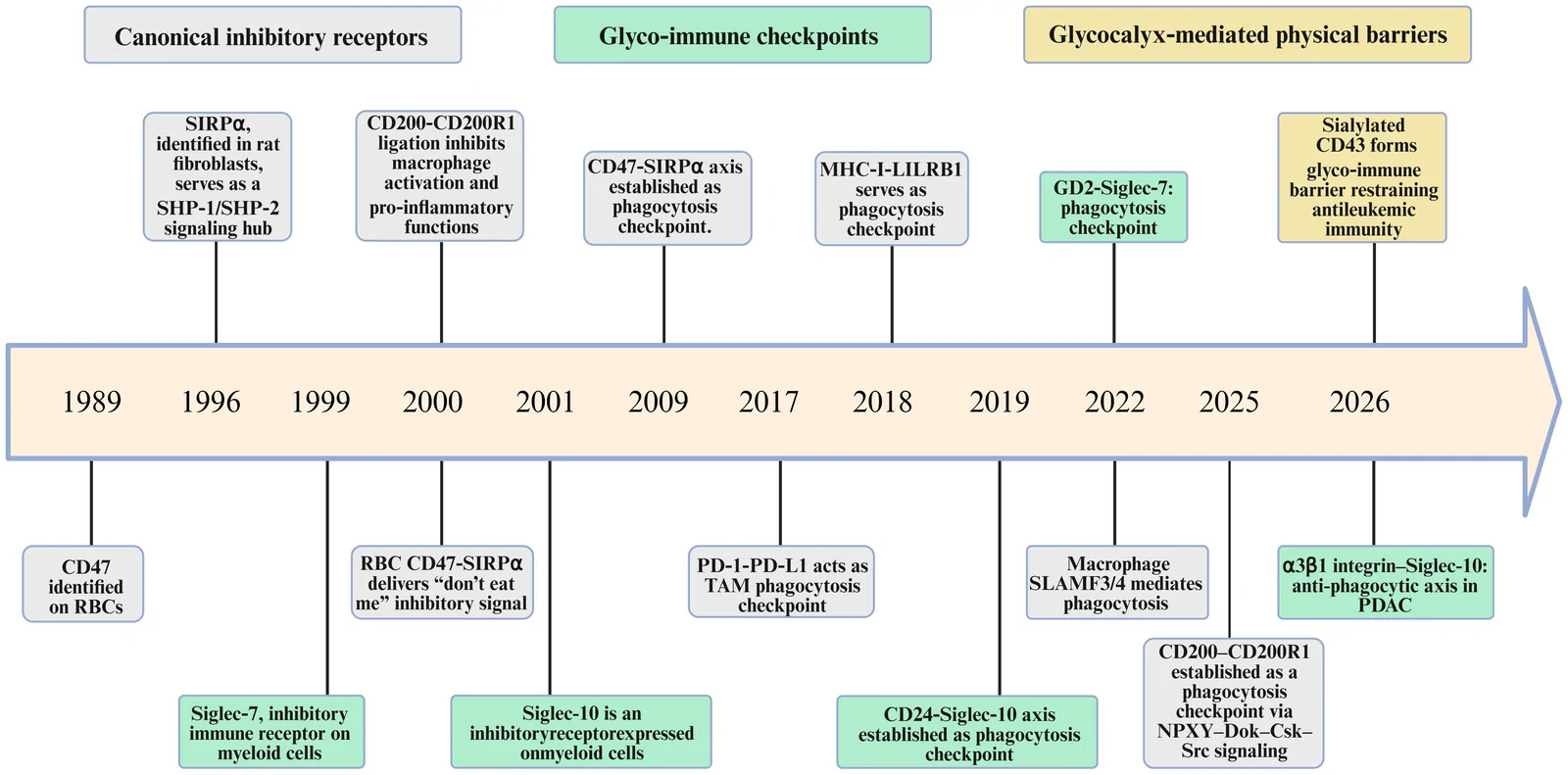
Evolution and classification of inhibitory phagocytosis checkpoints. Major milestones in the discovery of anti-phagocytic mechanisms are summarized chronologically, highlighting three emerging categories: canonical inhibitory receptors, glyco-immune checkpoints, and glycocalyx-mediated physical barriers. Early discoveries of CD47–SIRPα established the “don’t-eat-me” paradigm, whereas subsequent identification of additional inhibitory receptors, glycan-dependent checkpoints, and glycocalyx-associated barriers expanded the conceptual framework of tumor immune evasion. Created in BioRender. ding, M. (2026) https://BioRender.com/hbau0hr.

**Figure 2:**
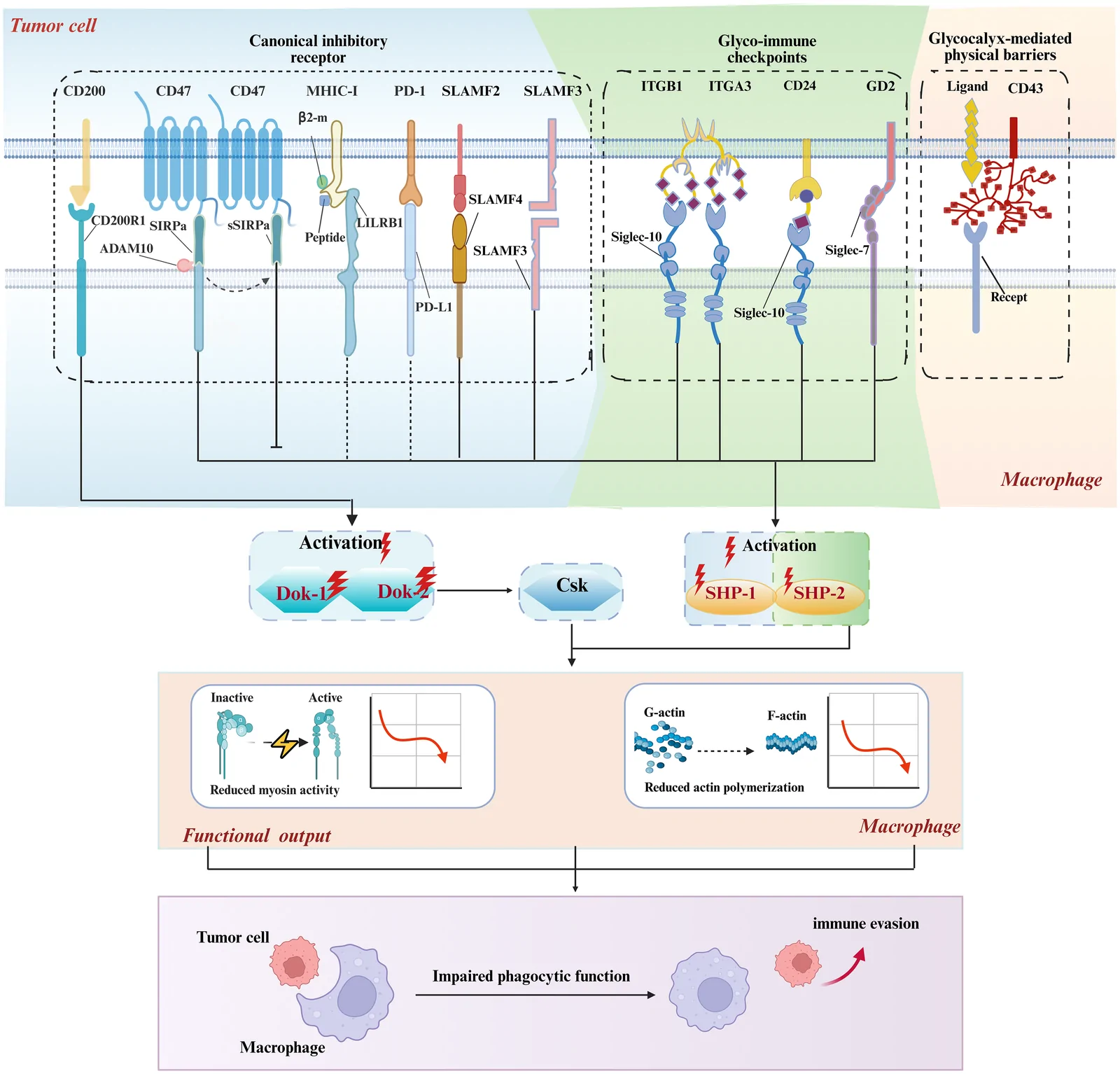
Mechanistic overview of tumor-mediated anti-phagocytic mechanisms and macrophage phagocytosis inhibition. Tumor cells evade macrophage-mediated clearance through multiple mechanistically distinct but functionally convergent anti-phagocytic pathways. Canonical receptor–ligand interactions, including CD47–SIRPα, MHC-I–LILRB1, PD-L1–PD-1 and CD200–CD200R1, transmit inhibitory signals that suppress macrophage activation and cytoskeletal remodeling. CD47–SIRPα signaling inhibits myosin activity, whereas CD200–CD200R1 primarily engages the Dok-1/Dok-2–Csk regulatory pathway. ADAM10-mediated ectodomain shedding of SIRPα generates soluble SIRPα (sSIRPα), modulate membrane-bound SIRPα signaling through competitive CD47 binding. SLAMF2–SLAMF4 and homophilic SLAMF3–SLAMF3 interactions can similarly generate inhibitory signals that restrain macrophage phagocytosis. Siglec-dependent glyco-inhibitory checkpoints include sialylated CD24–Siglec-10, α3β1 integrin–Siglec-10 and GD2–Siglec-7 interactions. Engagement of inhibitory Siglecs recruits SHP-1 and/or SHP-2 and attenuates downstream pathways required for macrophage activation and actin polymerization. Highly glycosylated and sialylated CD43 forms a noncanonical glycocalyx-mediated physical barrier that limits macrophage–tumor cell contact and phagocytic synapse formation through steric and electrostatic effects rather than canonical intracellular inhibitory signaling. ADAM10, a disintegrin and metalloproteinase 10; Csk, C-terminal Src kinase; Dok-1/2, docking protein 1/2; GD2, disialoganglioside GD2; ITGA3, integrin subunit α3; ITGB1, integrin subunit β1; LILRB1, leukocyte immunoglobulin-like receptor subfamily B member 1; MHC-I, major histocompatibility complex class I; β2m, β2-microglobulin; PD-1, programmed cell death protein 1; PD-L1, programmed cell death ligand 1; SHP-1, Src homology 2 domain-containing phosphatase 1; SHP-2, Src homology 2 domain-containing phosphatase 2; Siglec, sialic acid-binding immunoglobulin-like lectin; SIRPα, signal regulatory protein α; sSIRPα, soluble SIRPα; SLAMF, signaling lymphocytic activation molecule family. Created in BioRender. ding, M. (2026) https://BioRender.com/g5uys8s.

## 3 Canonical inhibitory receptor

### 3.1 The CD47–SIRPα axis

#### 3.1.1 Structure and physiological function

SIRPα, also known as SHPS-1, was one of the first SIRP family members to be identified (18). It is expressed primarily in myeloid immune cells (19), including nearly all macrophage subsets (20, 21), with lower expression reported in endothelial cells and selected central nervous system cells, such as microglia and neurons (22, 23). SIRPα is a single-pass transmembrane receptor whose extracellular region comprises one ligand-binding immunoglobulin variable (IgV)-like domain and two immunoglobulin constant (IgC)-like domains, whereas its cytoplasmic tail contains immunoreceptor tyrosine-based inhibitory motifs (ITIMs) (18, 24).

Upon ligand engagement, the SIRPα ITIMs become phosphorylated and recruit SH2 domain-containing protein tyrosine phosphatases, primarily SHP-1 and SHP-2, thereby initiating inhibitory signaling (25–27). This signaling suppresses myosin IIA activity and restricts cytoskeletal rearrangement and pseudopod formation, impairing phagocytic synapse formation and ultimately reducing macrophage phagocytosis and target-cell clearance (**Figure 2**) (25, 28). ADAM10-mediated ectodomain shedding can also generate soluble SIRPα that lacks intracellular signaling capacity and may modulate membrane-bound SIRPα signaling through competitive ligand binding (**Figure 2**) (21, 29, 30).

CD47 was originally identified as integrin-associated protein (IAP) (31). This broadly expressed cell-surface protein is the canonical ligand for SIRPα (32). Its extracellular IgV-like domain binds SIRPα on macrophages and delivers the classical “don’t eat me” signal (5). Unlike most single-pass immune receptors, CD47 has a distinctive five-transmembrane architecture and a variable cytoplasmic tail, features that contribute to its tissue-dependent functions (33, 34). Alternative splicing produces additional CD47 isoforms with distinct tissue distributions (35–37). In normal tissues, CD47–SIRPα engagement provides an inhibitory self-recognition signal that prevents inappropriate macrophage-mediated clearance and helps maintain tissue homeostasis.

#### 3.1.2 Functional hijacking in tumors

CD47 is frequently upregulated across diverse tumor types, and elevated expression has been linked to tumor progression, greater aggressiveness, and unfavorable clinical outcomes (38, 39). By sustaining CD47–SIRPα signaling, tumor cells deliver a “don’t eat me” signal to tumor-associated macrophages (TAMs) and co-opt the physiological self-recognition program to evade innate immune-mediated phagocytic clearance (40, 41). Blockade of the CD47–SIRPα axis enhances macrophage phagocytosis across multiple models of solid and hematologic malignancies (39, 42, 43).

Tumor cells can further reinforce this inhibitory axis through transcriptional, epigenetic, and post-translational mechanisms. IRF-1-mediated transcriptional activation, LSD1-associated epigenetic regulation, and ERK–MAN1B1-associated glycosylation can increase CD47 expression or enhance CD47–SIRPα interactions (44–46). Although operating at distinct regulatory levels, these mechanisms converge to increase tumor resistance to macrophage phagocytosis.

The CD47–SIRPα axis is essential for self-recognition and tissue homeostasis but is also co-opted by tumor cells to establish an anti-phagocytic microenvironment and evade immune clearance. This dual role both provides the mechanistic basis for therapeutic targeting and creates an inherent translational dilemma: relieving inhibitory signaling through this axis may increase tumor-cell susceptibility to phagocytosis, whereas the broad expression of CD47 on red blood cells(RBCs) and other normal cells can create an antigen sink, promote normal-tissue binding, and cause on-target/off-tumor toxicity (9–11). Relieving inhibitory signaling alone may be insufficient to induce effective phagocytosis in the absence of adequate pro-phagocytic drive. CD47-targeted drug design therefore requires optimization not only of blocking activity, but also of tumor selectivity, antibody format, Fc effector function, and the pro-phagocytic context.

#### 3.1.3 Therapeutic strategies and clinical translation

The CD47–SIRPα axis is one of the best-characterized inhibitory phagocytosis checkpoints and remains a central target in clinical development. Blockade of this pathway has been evaluated across multiple tumor types (**Table 1**). Based on their primary design objectives, CD47–SIRPα-targeted approaches can be broadly grouped into three categories: direct blockade of the CD47–SIRPα axis to relieve inhibitory signaling (6); engineered antibodies designed to improve tumor selectivity and hematologic safety through epitope selection, binding orientation, antibody valency, or modulation of Fc effector function (47, 48); and bispecific molecules or fusion proteins that enhance tumor targeting through tumor-antigen co-targeting or use SIRPα-derived CD47-binding domains to disrupt the axis (49, 50) (**Figure 3**). The clinical value of these approaches ultimately depends on achieving an appropriate balance among tumor exposure, pro-phagocytic activity, and normal-tissue safety.

**Table 1:** Clinical trial landscape of phagocytosis checkpoint blockade therapies.

Drug Type/Name	NCT trial identifier	Cancer type	Treatment type	Trial design	Current phase	Status
Anti-CD47 Antibody (AK117/ligufalimab)	NCT04980885	Acute Myeloid Leukemia	Alone or with Azacitidine	Dose escalation/Non- randomized	Phase 1/2	Active, not recruiting
	NCT06642792	Refractory Classic Hodgkin Lymphoma	Alone or with AK129	Dose escalation/Non- randomized	Phase 1/2	Recruiting
	NCT06196203	Myelodysplastic Syndromes	With Azacitidine or placebo	Randomized, double-blind, placebo-controlled	Phase 2	Recruiting
Anti-CD47 Antibody (IMC-002)	NCT05276310	Advanced Solid Tumors	Alone	Dose escalation	Phase 1	Recruiting
Anti-CD47/​PD-1 Bifunctional Antibody (HX009)	NCT05189093	Relapsed/​Refractory Lymphoma	Alone	Dose escalation/Non- randomized	Phase 1/2	Recruiting
	NCT06708663	Advanced Solid Tumors	Plus IN10018 with or without standard chemotherapy	Dose escalation/Non- randomized	Phase 2a	Recruiting
Anti-CD47/CD20 Bifunctional Antibody (IMM0306/Amulirafusp alfa)	NCT07355283	Relapsed/​Refractory Follicular Lymphoma	With Lenalidomide	Randomized, controlled, double-blind,	Phase 3	Not yet recruiting
SIRPα-Fc fusion protein (Maplirpacept)	NCT05675449	Multiple Myeloma	Elranatamab Plus Carfilzomib and Dexamethasone or Maplirpacept	Dose escalation	Phase 1b	Recruiting
SIRPα-Fc fusion protein (HCB101)	NCT05892718	advanced solid tumors or relapsed and refractory non-Hodgkin lymphoma	Alone	Dose escalation	Phase 1	Recruiting
SIRPα-Fc fusion protein (IMM01)	NCT07170787	Advanced Solid Tumors	Alone	Dose escalation	Phase 1	Not yet recruiting
	NCT05140811	Acute Myeloid Leukemia and Myelodysplastic Syndrome	With Azacitidine	Dose escalation/Non- randomized	Phase 1/2	Unknown status
SIRPα-Fc fusion protein (Evorpacept/ALX148)	NCT07007559	Advanced/​ Metastatic Malignancies	Combination With Anti-cancer Therapies	Dose escalation/Non- randomized	Phase 1b/2	Recruiting
SIRPα-Fc fusion protein (TTI-622)	NCT05507541	Relapsed or Refractory Diffuse Large B-Cell Lymphoma	With Pembrolizumab	Dose escalation/Non- randomized	Phase 2	Active, not recruiting
SIRPα-fused anti-PD-L1/TGF-β fusion protein (HCB301)	NCT06487624	advanced solid tumors or relapsed and refractory classical Hodgkin lymphomas	Alone	Dose escalation	Phase 1	Recruiting
Anti-LILRB1 Antibody (SAR444881/BND-22)	NCT06651593	Solid Tumors	Alone or with cemilipimab	Non- randomized	Phase 2	Recruiting
Anti-CD24 Antibody (IMM47)	NCT05985083	Advanced Solid Tumors	Alone	Dose escalation	Phase 1	Unknown status
Anti-CD24 Antibody (ATG-031)	NCT06028373	Advanced Solid Tumors or B-cell Non-Hodgkin Lymphomas	Alone	Dose escalation	Phase 1	Recruiting
Anti-CD24 Antibody (PHST001)	NCT06840886	Advanced Solid Tumors	Alone or in combination with chemotherapy	Dose escalation	Phase 1	Recruiting
Anti-CD24/​CD-3 Bifunctional Antibody (ONC-783)	NCT07408258	Advanced Solid Tumors	Alone	Dose escalation	Phase 1	Recruiting
Anti-Siglec-10 Antibody (ONC-841)	NCT06352359	Advanced Solid Tumors	Alone	Dose escalation	Phase 1	Recruiting
Peptide inhibitor(CD200AR-L)	NCT06305910	Recurrent high-grade glioma and newly diagnosed diffuse midline glioma/diffuse intrinsic pontine Glioma	Allogeneic tumor lysate vaccine immunotherapy with adjuvant reirradiation	Dose escalation	Phase 1	Recruiting

**Figure 3:**
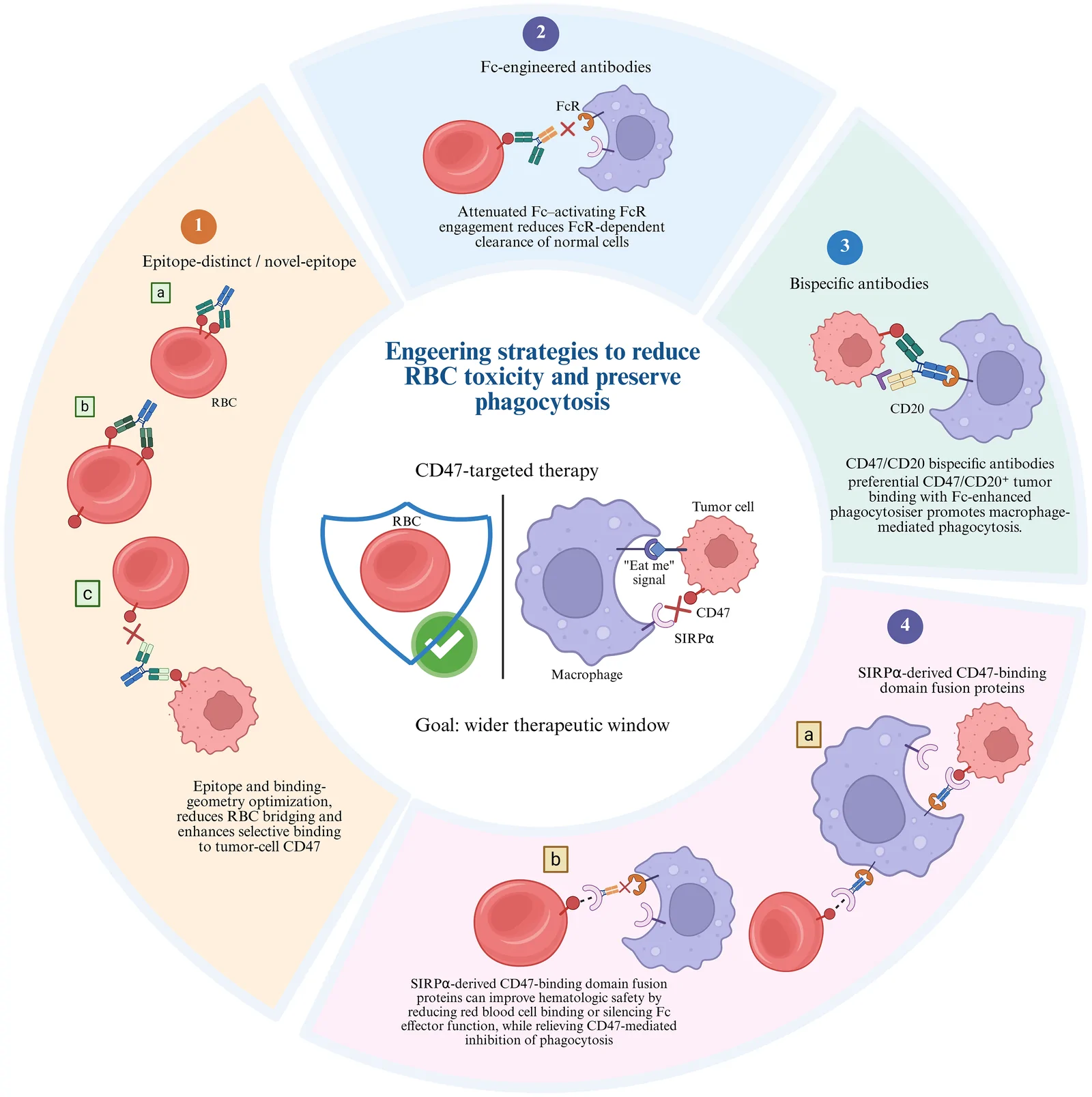
Engineering strategies to improve the therapeutic window of phagocytosis checkpoint therapies. Multiple engineering approaches have been developed to enhance tumor-selective phagocytic clearance while reducing hematologic toxicity associated with CD47–SIRPα pathway blockade. These strategies include epitope and binding-geometry optimization to minimize red blood cell (RBC) bridging and nonspecific binding, Fc engineering to attenuate Fcγ receptor (FcγR)-mediated clearance of normal cells, bispecific antibodies to preferentially redirect checkpoint blockade toward tumor cells, and SIRPα-derived CD47-binding fusion proteins to disrupt inhibitory signaling while modulating Fc effector activity. Created in BioRender. ding, M. (2026) https://BioRender.com/q00l0p3.

Before comparing different drug designs, it is important to distinguish RBC agglutination from accelerated phagocytic clearance. Agglutination arises primarily when the two Fab arms of a bivalent antibody bind CD47 on adjacent RBCs, creating RBC–antibody–RBC bridges that can propagate into larger aggregates (51). Accelerated phagocytic clearance, in contrast, results from disruption of CD47–SIRPα-mediated self-protective signaling, which increases RBC susceptibility to macrophage phagocytosis (5, 6); antibodies that retain FcγR binding can further promote FcγR-dependent antibody-mediated clearance (52) (**Figure 4**). RBC agglutination is typically a more acute and undesirable event but is not required for the development of anemia. Reducing intercellular cross-linking and agglutination therefore does not necessarily prevent RBC loss, and RBC binding, agglutination, and phagocytic clearance should be evaluated separately when assessing drug design.

**Figure 4:**
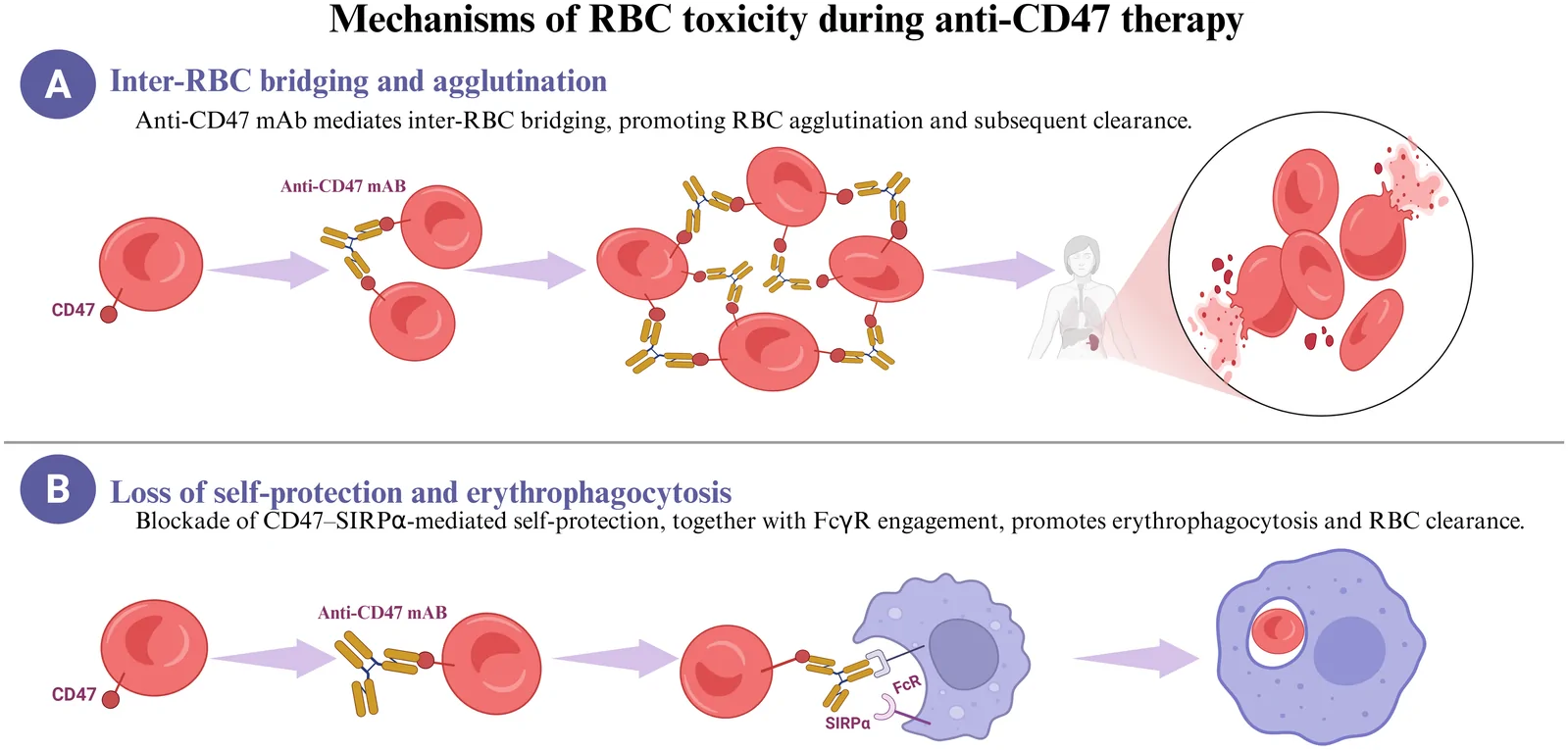
Mechanisms underlying hematologic toxicity associated with phagocytosis checkpoint blockade. Anti-CD47 therapies may induce red blood cell (RBC)-related toxicity through two mechanistically distinct pathways. **(A)** Inter-RBC bridging and agglutination. Bivalent anti-CD47 antibodies can simultaneously engage CD47 molecules on adjacent RBCs, forming RBC–antibody–RBC bridges that promote RBC aggregation and subsequent clearance. **(B)** Loss of self-protection and erythrophagocytosis. Blockade of the CD47–SIRPα interaction disrupts the physiological “don’t eat me” signal, and Fcγ receptor (FcγR) engagement can further promote macrophage-mediated erythrophagocytosis and RBC clearance. Created in BioRender. ding, M. (2026) https://BioRender.com/5vr4981.

##### 3.1.3.1 First-generation anti-CD47 monoclonal antibodies and clinical lessons

###### Magrolimab (Hu5F9-G4)

Magrolimab was among the first anti-CD47 antibodies to enter clinical development (6). By blocking the CD47–SIRPα interaction, it removes the antiphagocytic “don’t eat me” signal and permits existing pro-phagocytic signals on tumor cells to drive macrophage-mediated phagocytosis (53, 54). Preclinical studies showed that magrolimab enhanced macrophage phagocytosis of tumor cells, providing a mechanistic basis for its subsequent clinical development as monotherapy and in combination regimens (55).

In the first-in-human phase I study, a 1 mg/kg priming dose followed by maintenance dosing was used to mitigate early RBC toxicity. Transient anemia nevertheless remained the most common treatment-related adverse event, occurring in approximately 57% of patients (6). Preliminary antitumor activity was also observed, but the small sample size and lack of a comparator limit these efficacy findings to an early clinical activity signal (6).

In a phase Ib combination study in acute myeloid leukemia (AML), magrolimab plus azacitidine was associated with grade ≥3 anemia in 34.5% of patients, while the complete remission (CR) rate among patients with TP53-mutated disease was 31.9% (56). Many patients were already anemic at enrollment, indicating that underlying disease and bone marrow reserve may contribute to the observed hematologic toxicity (56). Disruption of CD47–SIRPα-mediated self-protective signaling can also increase RBC clearance, suggesting that target-related erythrocyte clearance may further contribute to treatment-emergent anemia (54). Some anemia occurred early during treatment and attenuated with continued dosing, but this pattern does not eliminate its potential constraint on the therapeutic window (56). Clinical activity was also observed with magrolimab plus rituximab in an early study of non-Hodgkin lymphoma (NHL) (57).

More importantly, subsequent phase III testing failed to demonstrate an overall survival benefit with magrolimab plus azacitidine in patients with TP53-mutated AML, and the trial was terminated early after meeting the prespecified futility criterion (11). In patients with AML who were ineligible for intensive therapy, the triplet of venetoclax, azacitidine, and magrolimab likewise failed to produce a significant improvement in clinical efficacy or survival outcomes (10).

The response signals observed in early nonrandomized studies therefore did not translate into survival benefits confirmed in randomized controlled trials, underscoring persistent uncertainty regarding both the clinical efficacy and therapeutic window of CD47 blockade and challenging the premise that efficacy can be improved through empirical drug addition. Combining CD47 blockade with tumor-associated antigen-targeting antibodies has a mechanistic rationale, as the latter can provide additional FcγR-mediated pro-phagocytic signaling (55). However, preclinical synergy and early clinical activity have not consistently translated into clinically meaningful incremental benefit. The development of magrolimab thus illustrates that early response rates are insufficient to predict survival benefit and that combination strategies should be guided by a defined need for additional pro-phagocytic signaling and demonstrable pathway dependence rather than by drug addition alone.

Magrolimab retains FcγR-binding capacity, which may support antibody-dependent phagocytosis of tumor cells but may also increase the risk of non-tumor cell clearance given the broad expression of CD47 on normal cells (6). The translational constraints imposed by Fc effector function, CD47 expression in normal tissues, and parallel inhibitory phagocytosis pathways are pathways are discussed further in Section 3.1.4.

SIRPα can also suppress CD18 activation through a cis interaction, providing a CD47-independent mechanism of phagocytic regulation (58). This finding suggests that concomitant disruption of the SIRPα–CD47 trans interaction and the SIRPα–CD18 cis interaction could further enhance phagocytosis (58). Whether an anti-SIRPα antibody can interfere with both interactions depends on its binding epitope and molecular format, and the generalizability and clinical relevance of this mechanism remain to be established.

##### 3.1.3.2 Epitope-distinct anti-CD47 monoclonal antibodies

Because CD47 is broadly expressed on normal cells, with particularly high expression on RBC (5, 59), CD47-targeting monoclonal antibodies face substantial on-target/off-tumor toxicity in clinical development, most commonly manifesting as early-onset anemia (53). Although priming-dose strategies can improve the manageability of these adverse events to some extent, they do not eliminate RBC-related toxicity (6). Therefore, subsequent anti-CD47 antibodies have been engineered primarily through optimization of epitope selection, binding orientation, antibody valency, and Fc effector function (**Figure 3**).

###### Lemzoparlimab (TJC4)

Lemzoparlimab is an epitope-distinct anti-CD47 monoclonal antibody that recognizes a unique CD47 epitope, thereby reducing RBC binding and the associated toxicity risk while retaining antitumor activity (47). In a phase Ib study of relapsed/refractory non-Hodgkin lymphoma (R/R NHL), lemzoparlimab monotherapy achieved an objective response rate (ORR) of 57% and a disease control rate (DCR) of 100%. No priming dose was required, and only limited mild hematologic toxicities were reported, most of which required no intervention (60). In a phase II study of lemzoparlimab plus azacitidine (AZA) in patients with intermediate- or high-risk myelodysplastic syndromes (MDS) by IPSS-R (61), preliminary results showed an ORR of 86.2% (25/29) and a CR rate of 31%. The most common grade 3/4 treatment-related adverse events remained hematologic, including decreased platelet, neutrophil, and white blood cell counts and anemia, with an overall safety profile broadly consistent with the known toxicity profile of AZA monotherapy (61). Public registry information indicates that the corresponding phase III study (NCT05709093) has been terminated (**Table 1**), leaving the early response signals and potential safety advantage unconfirmed in a later-phase randomized trial.

Overall, the epitope-distinct design of lemzoparlimab provides an engineering rationale for reducing RBC binding, but its clinical efficacy, hematologic safety, and value in combination therapy remain uncertain.

###### Ligufalimab (AK117)

AK117 is a humanized IgG4 anti-CD47 monoclonal antibody engineered through epitope selection and optimization of binding orientation to reduce intercellular RBC bridging and agglutination during CD47-targeted therapy (48). Mechanistic studies showed that Hu5F9-G4 adopts a head-to-head binding configuration with CD47, a geometry that favors formation of RBC–antibody–RBC bridges, promotes RBC aggregation, and may facilitate splenic retention and subsequent clearance of aggregated RBCs (**Figure 4**) (48, 51). By contrast, AK117 preferentially engages CD47 in a more lateral orientation with a smaller binding angle, making simultaneous binding to CD47 on adjacent RBCs less favorable and thereby reducing intercellular bridging and RBC agglutination (48).

Preclinical studies showed that AK117, either as monotherapy or in combination regimens, enhanced macrophage-mediated phagocytosis of B-cell lymphoma, T-cell leukemia, and Burkitt lymphoma cell lines, and demonstrated antitumor activity in xenograft models (48). Combination with the PD-1/VEGF bispecific antibody AK112 further increased macrophage phagocytosis and tumor-growth inhibition (48).

In a phase Ib study of newly diagnosed AML in patients ineligible for intensive therapy, AK117 plus AZA was associated with an anemia rate of 32.5% at a median follow-up of 6.7 months, while the composite CR rate among evaluable patients was 55% (CR 45%, CRi 10%) (62). Another early study of AK117 plus AZA in treatment-naive high-risk MDS reported a CR rate of 48.1%, an overall anemia rate of 30.6%, grade ≥3 anemia in 22.2% of patients, and RBC transfusion independence in 61.5% (63). These findings come from nonrandomized early-phase combination studies and require confirmation in larger randomized trials (62, 63). A phase II study of AK117 plus AZA in newly diagnosed high-risk MDS (NCT06196203) is ongoing (**Table 1**).

By optimizing the CD47-binding epitope and spatial orientation, AK117 reduces the likelihood of simultaneously engaging CD47 on separate RBCs and thereby limits intercellular bridging and agglutination (48). This structural optimization, however, does not fully eliminate anemia associated with CD47-targeted therapy. Even in the absence of overt RBC bridging, disruption of CD47–SIRPα-mediated self-protective signaling may still increase phagocytic clearance of RBCs. In addition, the reported anemia rates reflect overall hematologic outcomes during combination therapy and may also be influenced by pre-existing anemia, ineffective hematopoiesis, limited bone marrow reserve, and AZA-associated myelosuppression in patients with AML or MDS (63).

##### 3.1.3.3 Dual-epitope antibodies

Conventional bivalent anti-CD47 antibodies recognize the same epitope through both Fab arms, and their bivalent binding configuration may promote intercellular bridging between CD47 molecules expressed on adjacent cells (51, 64)(**Figure 4**). In hCD47 knock-in and hCD47/SIRPα double knock-in mouse models, Hu5F9 treatment significantly reduced RBC counts and hemoglobin levels (51). By contrast, the monovalent Hu5F9/gp120 format, which is less capable of inducing such bridging, showed no obvious agglutination across multiple experimental systems and caused only mild anemia in hCD47/SIRPα double knock-in mice (51).

A dual-epitope antibody, TJH2201, was subsequently developed to reduce the likelihood of antibody-mediated bridging between CD47 molecules on adjacent cell membranes (**Figure 3**) (51). In B-cell lymphoma and AML models, TJH2201 exhibited comparable CD47-blocking and pro-phagocytic activity to Hu5F9 and induced dose-dependent antitumor effects. Compared with Hu5F9, TJH2201 induced less RBC aggregation in the tested preclinical models and did not produce apparent anemia, suggesting that dual-epitope configuration and RBC-binding characteristics may reduce bridging-associated hematologic toxicity (51).

Importantly, reducing RBC bridging does not completely eliminate the risk of anemia. Monovalent CD47-binding molecules can still induce mild anemia in the absence of overt RBC agglutination (51), indicating that agglutination is not a prerequisite for CD47-targeted therapy-associated anemia. Phagocytic clearance resulting from disruption of CD47–SIRPα-mediated self-protective signaling may remain an independent contributor to RBC loss. The preclinical findings with TJH2201 support the feasibility of reducing certain hematologic toxicities through optimization of epitope configuration, antibody geometry, and RBC-binding properties, but do not establish complete separation between CD47-blocking activity and target-related toxicity. Its therapeutic window and clinical utility in CD47-positive malignancies, particularly hematologic cancers, require further clinical validation.

##### 3.1.3.4 Fc-engineered antibodies

Attenuating the interaction between the Fc region and activating FcγRs can reduce FcγR-dependent clearance of normal cells, particularly RBCs (65, 66). On this basis, Fc engineering has been used to mitigate on-target/off-tumor toxicity associated with CD47-targeted antibodies, especially RBC-related toxicity (**Figure 3**).

CC-90002 incorporates S228P and L235E substitutions in the IgG4 Fc region to attenuate effector function while preserving blockade of the CD47–SIRPα axis (66). This design reduces FcγR-dependent antibody-dependent cellular phagocytosis (ADCP) and the risk of phagocytic clearance of normal RBCs (66). Preclinical studies showed that CC-90002 enhanced macrophage phagocytosis of multiple hematologic and solid tumor cells and exerted antitumor activity across several xenograft models (67).

In a phase I study, however, dose escalation of CC-90002 monotherapy in patients with relapsed/refractory AML or high-risk MDS was terminated early because of limited single-agent activity and the development of anti-drug antibodies (68). Anemia occurred in 32.1% of patients, including grade 3–4 anemia in 28.6% (68). In R/R NHL, CC-90002 plus rituximab produced an ORR of 13%; hematologic toxicities remained the predominant adverse events, occurring in 50% of patients overall and at grade ≥3 in 13% (69).

Attenuation of Fc effector function may reduce some FcγR-dependent hematologic toxicity but does not fully eliminate the risk of anemia. It may also compromise Fc-dependent immune clearance and the cooperative effects of combination therapy, thereby limiting overall efficacy. Balancing reduced RBC-related toxicity against preservation of sufficient antitumor phagocytic activity therefore remains a key challenge in the engineering of CD47-targeted antibodies.

##### 3.1.3.5 Bispecific antibodies

Bispecific antibodies (BsAbs) are designed to preserve CD47–SIRPα blockade while incorporating a second target to promote tumor enrichment, lineage-restricted targeting, or enhancement of phagocytosis-related signals, with the aim of improving selective tumor-cell clearance while limiting systemic toxicity (**Figure 3**) (70, 71). Most of these strategies remain in preclinical or early clinical development.

###### 3.1.3.5.1 Tumor-associated antigen (TAA)–CD47 co-targeting

####### CD20-CD47 SL

First reported in 2015, the CD20–CD47 bispecific antibody CD20-CD47 SL uses a dual-variable-domain immunoglobulin (DVD-Ig) format in which two distinct variable domains are arranged in tandem at the N terminus of each heavy and light chain (72). The anti-CD20 variable domains are positioned distally at the N terminus, whereas the anti-CD47 domains lie closer to the constant regions. This configuration combines blockade of CD47–SIRPα inhibitory signaling with CD20-mediated enrichment on CD20-positive tumor cells, thereby enhancing macrophage-mediated phagocytic clearance (72). In co-cultures of Raji tumor cells and RBCs, CD20-CD47 SL preferentially bound CD20/CD47-double-positive tumor cells. In both localized and disseminated NHL xenograft models, CD20-CD47 SL monotherapy recapitulated the antitumor effect achieved by combined anti-CD47 antibody and rituximab treatment (72).

####### CC-96673

Building on tumor-antigen co-targeting, subsequent molecular designs have incorporated affinity and valency engineering to improve tumor selectivity and reduce hematologic toxicity. The CD47/CD20 bispecific antibody CC-96673, for example, uses an asymmetric affinity design with reduced affinity of the CD47-binding arm, thereby favoring engagement of CD47/CD20-double-positive tumor cells while limiting nonspecific binding to CD47 on RBCs and the associated hematologic toxicity (73). The antibody also retains IgG1 Fc effector function and can mediate antitumor activity through multiple Fc-dependent mechanisms, including antibody-dependent cellular cytotoxicity (ADCC), ADCP, and complement-dependent cytotoxicity (CDC) (73) (**Figure 3**). More recently, the 2 + 1-format CD38/CD47 bispecific antibody ISB 1442 showed greater antitumor activity than corresponding single-target controls in preclinical models of multiple myeloma (74).

####### HMBD004

The CD47/CD33 bispecific antibody HMBD004 uses the myeloid lineage marker CD33 to achieve lineage-restricted targeting of AML cells while blocking CD47–SIRPα signaling and enhancing selective phagocytosis. In mouse models of AML, HMBD004 showed antitumor activity and prolonged survival (75).

The rationale for TAA–CD47 co-targeting is to use highly expressed tumor antigens as anchoring targets, thereby concentrating CD47 blockade preferentially on tumor cells and reducing nonspecific engagement of CD47 on normal cells and the associated antigen-sink effect. Preclinical studies indicate that this approach can improve tumor selectivity and enhance antitumor activity in selected models; whether it can reduce RBC-related toxicity, widen the therapeutic window, and provide clinically meaningful benefit over single-target approaches remains to be established in patients.

###### 3.1.3.5.2 Immune checkpoint–CD47 co-targeting

Beyond tumor-antigen-directed approaches, some CD47-targeted bispecific molecules have been designed to concomitantly interfere with the PD-1/PD-L1 immunosuppressive pathway (49, 76). This strategy provides a mechanistic rationale for modulating distinct immune effector compartments, although whether multi-checkpoint blockade offers incremental benefit over monotherapy or conventional combination regimens remains uncertain.

####### HX009

HX009 was the first bispecific antibody developed to target both PD-1 and CD47 (76). It uses a symmetric 2×2 IgG4-based format that combines high-affinity PD-1 binding with low-affinity SIRPα-derived CD47 engagement, enhancing CD47 blockade while limiting nonspecific binding to RBCs and platelets and reducing the antigen-sink effect associated with widespread CD47 expression (76). In preclinical lymphoma models, HX009 showed greater antitumor activity than the corresponding single-target molecules, supporting its potential to modulate both innate and adaptive immunity (76).

In a phase Ib study of relapsed/refractory EBV-positive NHL, grade ≥3 neutropenia, leukopenia, lymphopenia, and anemia each occurred in 14.3% of patients, with no treatment-related deaths (77). Among 13 evaluable patients, the partial response rate was 23.1% and the disease control rate was 53.8% (77). In a separate study of advanced melanoma previously treated with immune checkpoint inhibitors, the objective response rate was 17.6% and the disease control rate was 52.9% among 17 evaluable patients (78). Given the small, uncontrolled cohorts and the early-phase nature of these studies, these findings should be regarded as preliminary clinical activity signals. Ongoing phase II studies are further evaluating the efficacy and safety of HX009.

####### 6MW3211

6MW3211 is another CD47/PD-L1 bispecific antibody (49), featuring an asymmetric design with high-affinity PD-L1 binding and low-affinity CD47 engagement. Recognition of CD47 by its CD47-binding arm is associated with N-linked glycosylation at N32 and N55, which may contribute to preferential tumor-cell binding and reduced RBC engagement (49). 6MW3211 simultaneously blocks the PD-1–PD-L1 and CD47–SIRPα pathways and enhances macrophage phagocytosis of PD-L1/CD47-double-positive tumor cells. In the MC38-hPD-L1/hCD47 model, treatment also increased intratumoral CD45^+^ immune-cell and CD8^+^ T-cell infiltration (49). This design illustrates an approach to modulating both macrophage phagocytosis and T-cell-associated immunity, although its clinical advantage remains to be established.

The principal rationale for CD47-related BsAbs is to preserve CD47–SIRPα blockade while incorporating a second target to promote tumor enrichment, lineage-restricted targeting, or coordinated modulation of innate and adaptive immunity, with the aim of improving selective tumor-cell clearance while reducing RBC binding, antigen-sink effects, and related hematologic toxicity associated with widespread CD47 expression. Their optimization extends beyond simple dual-target combination and requires integrated engineering of affinity, valency, and Fc effector function. Therapeutic performance also depends on the strength of pro-phagocytic signaling, tumor-antigen expression, and the treatment context. Most CD47-related BsAbs remain in preclinical or early clinical development (**Table 2**). Current evidence supports antitumor activity and the potential to improve tumor selectivity or safety, but whether these approaches can establish a durable efficacy–safety window in patients remains to be determined.

**Table 2:** Engineering strategies and translational design principles of phagocytosis checkpoint inhibitors.

Axis	Modality	Representative agent	Framework	Engineering strategy	RBC interaction	Clinical toxicity	Highest completed stage
CD47–SIRPα	Monoclonal antibody	Magrolimab (56)	IgG4 (53)	High-affinity Fab-mediated CD47 binding with retained FcγR engagement (53)	RBC opsonization, intercellular bridging, and hemagglutination (53)	Significant hematologic toxicity risk (10)	Phase 3
		Lemzoparlimab (47)	IgG4 (47)	Recognition of a distinct CD47 epitope with retained FcγR engagement (47)	Reduced RBC binding (47)	RBC-sparing profile; early studies suggest lower hematologic toxicity and no need for a priming dose (61)	Phase 2
		Ligufalimab (48)	IgG4 (48)	Recognition of a distinct CD47 epitope with optimized binding geometry and retained FcγR engagement (48)	Reduced intercellular bridging and RBC agglutination (48)	Engineered to minimize hemagglutination; improved hematologic safety reported (63)	Phase 1b
	Biparatopic antibody	TJH2201 (51)	IgG4 (51)	Biparatopic Fab arms recognizing two distinct CD47 epitopes with retained FcγR engagement (51)	Preferential dual-site engagement on the same cell surface, reducing intercellular bridging (51)	Reduced RBC toxicity in preclinical studies (51)	Preclinical Study
	Fc-engineered antibody	CC-90002 (66)	IgG4 (66)	High-affinity Fab-mediated CD47 binding with S228P/L235E Fc mutations to attenuate Fc effector function (66, 67)	Reduced RBC agglutination (66, 67)	Predominantly hematologic toxicity; overall safety manageable (66, 67)	Phase 1
	Bispecific antibody	CD20-CD47 SL (72)	IgG1 (72)	DVD-Ig format with tandem CD20- and CD47-targeting variable domains; retained FcγR engagement (72)	Preferential binding to CD20/CD47 double-positive tumor cells, reducing RBC binding (72)	Low risk of RBC-related toxicity in preclinical studies (72)	Preclinical Study
		CC-96673 (73)	IgG1 (73)	Separate Fab arms targeting CD20 and CD47, with attenuated CD47 affinity and retained FcγR engagement (73)	Preferential binding to CD20/CD47 double-positive tumor cells, reducing RBC binding (73)	No evident RBC binding in preclinical studies; favorable toxicity profile in non-human primates (73)	Preclinical Study
		HX009 (76)	IgG4 (76)	IgG4-based 2×2 anti-PD-1/CD47 bispecific incorporating an Fc-terminal low-affinity SIRPα extracellular domain, with retained FcγR engagement (76)	Preferential binding to PD-1/CD47 double-positive cells (76)	Generally well tolerated; mainly manageable hematologic toxicity; no treatment-related deaths reported (76, 77)	Phase 1b
	SIRPα-binding domain-containing fusion proteins	IMM01 (50)	IgG1 (50)	SIRPα D1–human IgG1 Fc fusion protein with retained FcγR engagement (50)	Minimal RBC binding (50)	Well tolerated; grade ≥3 hemolysis rare (79)	Phase 2
		nFD164 (80)	IgG1 (80)	Fc-silenced SIRPα D1–human IgG1 Fc fusion protein (80)	Minimal RBC binding (80)	No RBC agglutination *in vitro*; no significant RBC decline *in vivo* (80)	Preclinical Study
MHC-I–LILRB1	Monoclonal antibody	BND-22(SAR444881) (81)	IgG4 (81)	High-affinity Fab-mediated LILRB1 binding with retained FcγR engagement (81)	No RBC binding (81)	No treatment-related dose-limiting toxicity observed in phase I (82)	Phase 1
		LILRB1-IgGσ (83)	IgG2 (83)	High-affinity Fab-mediated LILRB1 binding; Fc-silent (83)	No RBC binding (83)	No apparent toxicity *in vitro* or *ex vivo* (83)	Preclinical Study
CD24-Siglec-10	Monoclonal antibody	IMM47 (84)	IgG1 (84)	High-affinity Fab-mediated CD24 binding with retained Fc-mediated effector function (84)	No RBC binding (84)	No significant toxicity observed in preclinical studies (84)	Preclinical Study
	Bispecific antibody	CD24/Siglec-10 blocking peptide (85)	Peptide (85)	Cross-target recognition enabled by structural similarity between CD24 and PD-L1 (85)	No RBC binding (85)	No significant toxicity observed in preclinical studies (85)	Preclinical Study
	Oncolytic adenovirus	AdV-mSS (86)	Adenovirus type 5 backbone (86)	Ad5-based construct encoding a secreted mSIRPα/mSiglec-G dual-ECD fusion protein (86)	Intratumoral delivery may mitigate hemolysis risk associated with systemic CD47 blockade (86)	No significant toxicity observed in preclinical studies (86)	Preclinical Study

##### 3.1.3.6 SIRPα-derived CD47-binding domain fusion proteins

SIRPα-derived CD47-binding domain fusion proteins are engineered biologics generated by fusing the CD47-binding domain of SIRPα with additional functional modules, such as Fc regions. These molecules competitively disrupt CD47–SIRPα interactions, relieve phagocytic inhibitory signaling, and enhance macrophage-mediated tumor-cell phagocytosis (50, 87).

###### IMM01

IMM01 is a recombinant SIRPα-Fc fusion protein consisting of the extracellular domain of SIRPα fused to a human IgG Fc fragment (50). It binds CD47 on tumor cells and competitively disrupts CD47–SIRPα interactions, thereby relieving CD47–SIRPα-mediated phagocytic inhibition (50). *In vitro* studies showed minimal binding of IMM01 to human RBCs and no detectable induction of RBC aggregation or phagocytosis. This feature may be related to the influence of RBC-specific CD47 glycosylation patterns on SIRPα binding and may contribute to improved hematologic safety (**Figure 3****) (**50).

Beyond relieving the “don’t eat me” signal, the Fc domain of IMM01 engages activating Fcγ receptors on macrophages, providing additional pro-phagocytic signals and enhancing ADCP. This creates a dual mechanism involving both blockade of phagocytic inhibition and reinforcement of phagocytic activation (50, 88). Preclinical studies demonstrated antitumor activity of IMM01 monotherapy in hematologic malignancies, with additional potential in combination settings for selected solid tumor models (50). In a phase II study of high-risk MDS, IMM01 combined with azacitidine achieved an ORR of 64.7% and a CR rate of 29.4%; anemia accounted for 43.9% of all grade ≥3 treatment-related adverse events (TRAEs) (79). Although preclinical studies did not show substantial RBC binding, aggregation, or phagocytosis induced by IMM01, the occurrence of anemia in clinical studies suggests that hematologic toxicity may be influenced by underlying disease status, combination regimens, and non-aggregation-related mechanisms. Further studies are required to define its safety profile and mechanism of action.

###### nFD164

nFD164 is an Fc-silenced SIRPα–Fc fusion protein comprising a modified SIRPα V1 domain fused to an IgG1 Fc region containing the N297A mutation to attenuate Fc-mediated effector function (80, 89). In preclinical studies, nFD164 caused no marked abnormalities in RBC, platelets, or leukocytes and showed modest single-agent antitumor activity, with enhanced effects when combined with immune checkpoint inhibitors or targeted antibodies (80). These findings support the feasibility of improving hematologic safety through Fc attenuation, although its clinical efficacy and optimal combination strategy remain to be established.

A key design consideration for SIRPα fusion proteins is the balance between retained Fc effector function and CD47 blockade(**Figure 3**). Different formats therefore involve distinct trade-offs among single-agent activity, combination potential, and hematologic safety, and their optimal use is likely to depend on tumor context and treatment setting.

#### 3.1.4 Translational limitations and unresolved problems

Current CD47-targeted agents use epitope and binding-orientation optimization, valency modulation, Fc engineering, and tumor-directed designs to reduce RBC bridging and agglutination, FcγR-dependent clearance, or nonspecific engagement of normal tissues (**Figure 3**) (47, 48, 66). These engineering strategies address distinct components of toxicity, and reducing one hematologic risk does not eliminate CD47-targeted treatment-related toxicity as a whole. For example, limiting RBC agglutination does not prevent phagocytic clearance following disruption of CD47-mediated self-protective signaling (51), whereas attenuation of Fc effector function may reduce ADCP of normal cells at the cost of impaired tumor-cell clearance (68). Baseline anemia, limited bone marrow reserve, and treatment-related myelosuppression may further influence hematologic outcomes. CD47-targeted drug design therefore continues to require a balance among tumor selectivity, pro-phagocytic activity, and normal-tissue safety.

Beyond the therapeutic window, the efficacy of CD47 blockade is shaped by the strength of pro-phagocytic signals, the FcγR expression and functional context, macrophage state, and parallel inhibitory phagocytosis pathways (68, 90, 91). Synergy or response signals observed with agents such as magrolimab in preclinical and early nonrandomized studies have not consistently translated into survival benefits confirmed in randomized trials, indicating that CD47 expression alone is insufficient for patient selection. Future studies should define the functional dependence of individual tumors on the CD47–SIRPα axis and develop stratification strategies that integrate tumor-antigen expression, pro-phagocytic signaling, and macrophage state. Multi-checkpoint blockade and other combination strategies should likewise be guided by mechanistic complementarity and comparative clinical evidence rather than by preclinical synergy alone.

### 3.2 MHC-I–LILRB1 axis

#### 3.2.1 Structure and physiological function

Major histocompatibility complex class I (MHC-I) molecules consist of a polymorphic transmembrane heavy chain noncovalently associated with a β2-microglobulin (β2m) light chain and are broadly expressed on nucleated cells (92, 93). The MHC-I heavy chain contains three extracellular domains, α1, α2, and α3. The α1 and α2 domains together form the peptide-binding groove, which binds and presents endogenous peptides for antigen-specific recognition by CD8^+^ T cells (94). The α3 domain and β2m contribute to the structural stability and cell-surface expression of MHC-I and support formation of a stable MHC-I heavy chain–β2m–peptide complex (95).

LILRB1 (also known as ILT2) is a single-pass inhibitory receptor expressed across multiple immune-cell populations, including macrophages (96), natural killer (NK) cells (97), and dendritic cells (DCs) (98), and is highly expressed in TAMs (16). Its extracellular region comprises four immunoglobulin-like domains (D1–D4) (99), with the N-terminal D1 and D2 domains forming the MHC-I-binding interface that primarily recognizes the α3 domain of the MHC-I heavy chain and β2m (100). MHC-I engagement induces phosphorylation of the cytoplasmic ITIMs and recruitment of the inhibitory phosphatases SHP-1 and SHP-2 (16, 101), thereby suppressing immune receptor-associated activating signals (**Figure 2**) (101). This ITIM-dependent signaling architecture creates partial functional convergence between the MHC-I–LILRB1 axis and other inhibitory phagocytosis checkpoints.

#### 3.2.2 Functional hijacking in tumor

Tumor regulation of MHC-I has dual immunological consequences. Downregulation of MHC-I can reduce presentation of tumor-derived antigens to CD8^+^ T cells and thereby weaken adaptive immune surveillance (102, 103). Conversely, in certain immunological and tumor-microenvironmental contexts, intact cell-surface MHC-I can be functionally co-opted as an inhibitory ligand for LILRB1 on myeloid cells, limiting macrophage-mediated phagocytic clearance (101, 104). Barkal et al. showed that the MHC-I expression state of tumor cells is associated with resistance to macrophage phagocytosis and that this effect depends on LILRB1 expressed by TAMs (16, 81). Disruption of the MHC-I–LILRB1 interaction relieves this inhibitory signal and enhances macrophage phagocytosis of tumor cells (16, 81).

The MHC-I–LILRB1 axis therefore illustrates a functional tension between adaptive and myeloid immunity: the same MHC-I molecule contributes to antigen recognition by CD8^+^ T cells (102, 103) while also providing an anti-phagocytic signal in the presence of LILRB1-positive myeloid cells (101, 104). Its functional importance is not determined by tumor MHC-I expression alone, but also by the abundance and state of local LILRB1-positive effector cells, the strength of pro-phagocytic signals, and the contribution of parallel inhibitory pathways. This context dependence suggests that future LILRB1-targeted strategies may require patient stratification based on both tumor-intrinsic and myeloid immune features.

#### 3.2.3 Therapeutic development and clinical translation

Therapeutic development of the MHC-I–LILRB1 axis has focused primarily on LILRB1 blockade to relieve suppression of myeloid-cell effector function. Barkal et al. showed that disruption of MHC-I–LILRB1 interactions using W6/32 Fab fragments or direct blockade with anti-LILRB1 antibodies enhanced macrophage phagocytosis of tumor cells, providing direct preclinical proof of concept for therapeutic targeting of this axis (16). Combined LILRB1 and CD47 blockade further increased phagocytosis in selected experimental models (16), suggesting that the MHC-I–LILRB1 and CD47–SIRPα axes can provide partially nonredundant inhibitory inputs in specific contexts. These findings remain preclinical, however, and do not establish that multi-checkpoint blockade provides clinically meaningful incremental benefit.

##### BND-22

BND-22 (SAR444881) is a humanized IgG4 monoclonal antibody targeting LILRB1 that relieves phagocytic inhibition by disrupting the LILRB1–MHC-I interaction (81). Preclinical studies showed antitumor activity across multiple solid tumor models, with further enhancement when combined with PD-1- or EGFR-targeted therapy (81). In a phase I study of patients with advanced solid tumors (NCT04717375), no treatment-related dose-limiting toxicities were observed with BND-22 either as monotherapy or in combination with pembrolizumab or cetuximab (82). Common treatment-related adverse events included fatigue, nausea, and rash. High target-receptor occupancy was achieved, and partial responses and disease control were observed, including in some patients expected to derive limited benefit from, or who had progressed after, prior anti-PD-1 and/or cetuximab therapy (82). However, this was primarily a dose- and safety-finding study with a limited sample size and no randomized comparator, precluding firm conclusions regarding the independent antitumor activity of LILRB1 blockade or any incremental benefit of the combination regimens. A phase II study of BND-22 in selected solid tumors (NCT06651593) is ongoing.

##### LILRB1-IgGσ

LILRB1-IgGσ is an Fc-silenced anti-LILRB1 monoclonal antibody (83). LILRB1 blockade alone was insufficient to substantially enhance macrophage phagocytosis, but phagocytosis of multiple lymphoma cell lines increased when LILRB1 blockade was combined with CD47 blockade and a CD20-targeting antibody (83). Sensitivity to this combination correlated with the CD20/HLA-I expression ratio: in cells with high HLA-I and low CD20 expression, rituximab alone or together with CD47 blockade induced limited phagocytosis, whereas additional LILRB1 blockade enhanced the response (83). These findings indicate that the effect of LILRB1 blockade depends on the balance between MHC-I–LILRB1-mediated inhibitory input and tumor-antigen/FcγR-dependent pro-phagocytic drive. Relief of LILRB1 inhibition alone may therefore be insufficient when pro-phagocytic signaling is weak, supporting treatment selection based on pathway dependence and phagocytic context rather than assuming an inherent advantage of multi-checkpoint blockade.

#### 3.2.4 Translational limitations and unresolved problems

A major challenge for therapeutic targeting of the MHC-I–LILRB1 axis is defining tumor contexts that are truly dependent on this pathway. Unlike CD47, MHC-I contributes both to antigen presentation to CD8^+^ T cells and to LILRB1-mediated myeloid inhibition (101, 102). High MHC-I expression therefore cannot be interpreted simply as a unidirectional immunosuppressive marker, whereas MHC-I downregulation may reduce LILRB1-mediated inhibitory input at the cost of impaired adaptive immune recognition. LILRB1 is also not restricted to macrophages but is expressed on multiple immune-cell populations, including NK cells and dendritic cells (97, 98), making it difficult to attribute the clinical effects of LILRB1 blockade solely to restoration of macrophage phagocytosis. Tumor MHC-I or LILRB1 expression alone is therefore unlikely to be sufficient for response prediction, as functional relevance also depends on ligand–receptor co-expression, local immune-cell composition, and spatial organization.

Preclinical findings further indicate that the effects of LILRB1 blockade depend strongly on the pro-phagocytic context (81, 83). Future patient stratification may therefore need to integrate tumor MHC-I/β2m status, the abundance and functional state of LILRB1-positive myeloid cells, tumor-antigen density, and FcγR-dependent pro-phagocytic capacity rather than relying on a single biomarker. Early clinical studies of agents such as BND-22 support the druggability of LILRB1 (82), but its independent therapeutic value, optimal treatment context, and predictive biomarkers remain undefined. Combination blockade of LILRB1 with CD47 or other inhibitory checkpoints should likewise be guided by demonstrable pathway co-dependence and mechanistic complementarity, with incremental benefit ultimately established in randomized controlled trials.

### 3.3 The PD-1–PD-L1 axis

#### 3.3.1 Structure and physiological function

Programmed cell death protein 1 (PD-1) was first identified in 1992 and was subsequently established as a key inhibitory receptor regulating T-cell activation (133). PD-1 is expressed on multiple immune-cell populations, including T cells, NK cells, and macrophages (133–135). It is a type I transmembrane protein with an extracellular immunoglobulin variable (IgV)-like domain involved in ligand recognition (136). Its cytoplasmic region contains both an ITIM and an immunoreceptor tyrosine-based switch motif (ITSM). Upon ligand engagement, these motifs become phosphorylated and predominantly recruit SHP-2, with SHP-1 also implicated in some settings, thereby attenuating downstream activating signals and constraining the magnitude and duration of T-cell responses (136, 137). Its major ligand, PD-L1, is broadly expressed on tumor cells and multiple immune-cell populations (134). Although the PD-1–PD-L1 pathway is best known as a canonical T-cell immune checkpoint, evidence also supports a role in regulating macrophage function in specific tumor microenvironments (**Figure 2****) (**135, 138).

#### 3.3.2 Functional hijacking in tumors and translational considerations

PD-1 expression has been detected on TAMs in selected murine tumor models and human tumor samples, with increased expression associated with tumor progression (135). PD-1^+^ TAMs frequently exhibit immunosuppressive features and impaired phagocytic capacity (135). In the relevant experimental models, disruption of PD-1–PD-L1 interactions or loss of tumor-cell PD-L1 restored phagocytic activity in PD-1^+^ TAMs, whereas phagocytosis by PD-1^−^ TAMs was less dependent on tumor-cell PD-L1 status (135).

The clinical antitumor activity of PD-1/PD-L1 blockade, however, cannot be attributed directly to restoration of macrophage phagocytosis because this pathway broadly regulates T cells and other immune effector populations. Current evidence more specifically supports a phagocytosis-regulatory role for PD-1 in selected PD-1^+^ TAM subsets rather than a universal anti-phagocytic mechanism across TAMs. The distribution and functional states of PD-1^+^ TAMs, as well as their independent contribution to clinical responses to PD-1/PD-L1 blockade, remain to be defined.

### 3.4 The CD200-CD200R1 axis

#### 3.4.1 Structure and physiological function

CD200, also known as OX-2, is a type I transmembrane glycoprotein belonging to theIgSF. It has a simple domain organization, consisting of an ECD, a TMD, and a short cytoplasmic domain (139, 140). CD200 is broadly expressed in normal tissues and is found on several immune and non-immune cell populations, including dendritic cells, neurons, endothelial cells, and intestinal mucosal cells (141–143). Through its extracellular Ig-like domain, CD200 binds CD200R1 and participates in the control of immune-cell activation (140).

CD200R1 is also an IgSF member and comprises two extracellular Ig-like domains, a transmembrane region, and a cytoplasmic tail (144). It is expressed on macrophages, microglia, T cells, and neutrophils (145–147). Unlike SIRPα, LILRB1, and inhibitory Siglecs, CD200R1 does not signal through the canonical ITIM–SHP pathway. Instead, its cytoplasmic tail contains several conserved tyrosine residues (Y286, Y289, and Y297), with Y297 located within a critical NPXY motif (14). Upon CD200 engagement, Y297 becomes phosphorylated, enabling the NPXY motif to recruit docking protein 1 and 2 (Dok-1/Dok-2) and propagate inhibitory signaling through negative regulators such as Ras GTPase-activating protein (Ras-GAP) (**Figure 2**) (14, 148). Dok-1 can also transiently associate with phosphorylated SH2-containing inositol 5′-phosphatase (SHIP), further attenuating downstream activating signals (148). Physiologically, the CD200–CD200R1 axis restrains excessive myeloid-cell activation and contributes to tissue immune homeostasis. In the nervous system, for example, neuronal CD200 engages CD200R1 on microglia to help maintain a relatively quiescent microglial state (149). This physiological inhibitory program also provides a mechanistic basis for tumor-cell co-option of the CD200–CD200R1 axis to suppress myeloid immune effector function.

#### 3.4.2 Functional hijacking in tumors and translational considerations

CD200 is upregulated in multiple tumor types and has been associated with immunosuppression and unfavorable clinical outcomes (150, 151). More direct evidence supports CD200–CD200R1 as a receptor–ligand axis that suppresses macrophage phagocytosis. Engagement of CD200R1 on macrophages by CD200 inhibits phagocytic signaling downstream of multiple pro-phagocytic receptors, including FcγR and signaling lymphocytic activation molecule family member 7 (SLAMF7) (14). Genetic disruption or monoclonal antibody blockade of this axis enhances macrophage phagocytosis of cancer cells and suppresses tumor growth in mice (14). Mechanistically, the NPXY motif of CD200R1 signals through Dok proteins to regulate C-terminal Src kinase (Csk) and suppress Src family kinase activity, thereby limiting early signaling downstream of multiple pro-phagocytic receptors (14, 152). Although CD200R1 uses a proximal inhibitory mechanism distinct from that of ITIM–SHP-1/SHP-2-dependent phagocytosis checkpoints, its functional output similarly converges on attenuation of pro-phagocytic signaling and restriction of tumor-cell clearance (**Figure 2**).

Beyond its direct effects on phagocytosis, tumor-derived CD200 can modulate additional immunosuppressive processes through CD200R1^+^ immune cells. In pancreatic cancer, CD200 promotes MDSC expansion and enhances their suppressive activity (150), whereas in multiple myeloma it impairs NK-cell function and attenuates daratumumab-dependent ADCC (151, 153). CD200 can also activate β-catenin/NF-κB/macrophage colony-stimulating factor (M-CSF) signaling and increase M-CSF secretion, promoting the development of immunosuppressive, tumor-promoting macrophage states in the tumor microenvironment of head and neck squamous cell carcinoma (154). These findings indicate that, beyond directly suppressing immune effector function, the CD200–CD200R1 axis may also contribute to tumor progression by reshaping macrophage states.

CD200–CD200R1 differs from CD47–SIRPα in both expression pattern and signaling mechanism, suggesting that the sources of toxicity and safety constraints associated with its blockade may also differ (14). This distinction, however, should not be interpreted as evidence of a broader therapeutic window. Current clinical evidence is insufficient to establish a more favorable efficacy–safety profile for CD200–CD200R1 blockade. The enhanced phagocytosis observed with combined CD200–CD200R1 and CD47–SIRPα blockade has so far been demonstrated only in preclinical settings (14) and does not establish clinical synergy or an improved efficacy–safety balance. The CD200–CD200R1 axis is therefore best regarded as an inhibitory phagocytosis checkpoint with direct preclinical evidence of phagocytic regulation but as-yet unestablished clinical therapeutic value.

### 3.5 SLAMF3 and SLAMF4

#### 3.5.1 Structure and physiological function

SLAMF3 (Ly9, CD229) and SLAMF4 (2B4, CD244) are type I transmembrane receptors of the CD2/SLAM subfamily of the IgSF and are expressed predominantly on hematopoietic cells (155–157). The extracellular region of SLAMF3 contains two tandem IgV–C2-like domain pairs that mediate homophilic SLAMF3–SLAMF3 interactions, whereas SLAMF4 contains one IgV-like and one C2-like domain and primarily recognizes its ligand SLAMF2 (CD48) (156, 158, 159). Both receptors contain ITSMs in their cytoplasmic tails, with two ITSMs in SLAMF3 and four in SLAMF4 (156, 160).

Signaling through SLAMF3 and SLAMF4 is highly context dependent. Following ITSM phosphorylation, distinct signaling complexes can form depending on the availability of SLAM-associated protein (SAP)-family adaptors and SH2 domain-containing phosphatases, resulting in either activating or inhibitory signaling (156, 161–163). In the context of macrophage phagocytosis, SLAMF3 and SLAMF4 can mediate SAP-independent inhibitory signaling, indicating that their functional output is determined by the intracellular signaling context (15).

#### 3.5.2 Functional hijacking in tumors and translational considerations

SLAMF3 and SLAMF4 were initially identified as inhibitory receptors that restrain macrophage phagocytosis in hematopoietic-cell contexts. SLAMF3 engages SLAMF3 on target cells through homophilic interactions, whereas SLAMF4 on macrophages recognizes SLAMF2 on target cells (156, 158, 159). These interactions generate SHP-1- and SHP-2-associated inhibitory signaling in macrophages and restrict activation of Syk and mechanistic target of rapamycin complex 1 (mTORC1) downstream of low-density lipoprotein receptor-related protein 1 (LRP1), thereby attenuating pro-phagocytic signaling (**Figure 2****) (**15). Genetic disruption or antibody-mediated interference with these SLAMF interactions enhances macrophage phagocytosis of hematologic tumor cells and promotes tumor clearance in mouse models (15). Although SLAMF3/4 differ from canonical ITIM-dependent checkpoints in their proximal signaling architecture, their functional output partially converges with that of other inhibitory phagocytosis pathways through suppression of Syk and related pro-phagocytic programs.

More recent work has extended SLAMF3-associated phagocytic inhibition to solid tumor settings. In a model of colorectal cancer liver metastasis, metastatic tumor cells exhibited increased SLAMF3 expression and greater resistance to phagocytosis, whereas SLAMF3 knockdown enhanced macrophage phagocytosis of tumor cells (164). SLAMF3 knockdown was also accompanied by changes in TAM composition and macrophage–CD8^+^ T-cell interactions, including an increased proportion of C1QC^+^ TAMs and fewer exhausted CD8^+^ T cells (164), suggesting that the effects of this pathway may extend beyond direct regulation of phagocytosis.

Current evidence supports SLAMF3 and SLAMF4 as macrophage anti-phagocytic receptors in selected hematologic malignancy contexts and suggests that SLAMF3-related mechanisms may also operate in some solid tumors. The evidence remains predominantly preclinical, however, and SLAMF receptor signaling is strongly context dependent. In mice, concurrent loss of SLAMF receptors and CD47 further relieved phagocytic inhibition but also caused more pronounced depletion of hematopoietic cells (15), suggesting that these anti-phagocytic pathways may contribute not only to tumor immune evasion but also to immune tolerance of normal hematopoietic cells. Their therapeutic value must therefore be evaluated in the context of normal hematopoietic homeostasis and the therapeutic window.

## 4 Glyco-inhibitory checkpoints

### 4.1 Siglec-dependent glyco-inhibitory checkpoints

Siglecs (sialic acid-binding immunoglobulin-like lectins) are type I transmembrane lectin receptors of the immunoglobulin superfamily (IgSF) that primarily recognize sialylated glycans on the cell surface; approximately 15 members have been identified in humans (105). These receptors are expressed predominantly on hematopoietic immune cells, including macrophages, dendritic cells, neutrophils, B cells, and selected T-cell subsets, where they contribute to immune homeostasis by monitoring cell-surface sialylation patterns (106–108). Their extracellular regions typically contain a V-set Ig-like domain that mediates ligand recognition (109). On the basis of their intracellular signaling capacity, Siglecs are commonly classified as activating or inhibitory receptors (110). Activating Siglecs generally associate, through positively charged residues in the transmembrane region, with ITAM-containing adaptor proteins (110). By contrast, inhibitory Siglecs, including Siglec-7 and Siglec-10, contain ITIMs in their cytoplasmic tails and recruit SHP-1 and/or SHP-2 phosphatases after ligand engagement, thereby limiting immune-cell activation and effector function (111, 112). Tumor cells can co-opt this inhibitory machinery by increasing or remodeling sialylated glycoproteins and glycolipids on their surface, thereby establishing glycosylation-dependent anti-phagocytic mechanisms (17, 113). Unlike CD47–SIRPα and MHC-I–LILRB1, which are governed largely by protein–protein recognition interfaces, Siglec-dependent inhibition is determined not only by ligand abundance but also by ligand glycosylation and sialylation state and the expression context of the relevant Siglec receptor.

#### 4.1.1 CD24–Siglec-10 axis

##### 4.1.1.1 Structure and physiological function

CD24 is a highly glycosylated glycosylphosphatidylinositol (GPI)-anchored membrane protein expressed on subsets of immune and non-hematopoietic cells, with expression patterns dependent on cell type and differentiation state (114, 115). The mature protein contains a short polypeptide core of only 31–35 amino acids and is composed of the peptide backbone, extensive glycosylation modifications, and a GPI anchor (116). CD24 contains multiple glycosylation sites, and its glycan composition and sialylation status influence membrane localization, ligand recognition, and immunoregulatory functions (117–119).

##### 4.1.1.2 Functional hijacking in tumors and translational considerations

CD24 is frequently overexpressed in multiple tumor types, and its sialylated glycans provide a key molecular basis for recognition by Siglec-10 on macrophages (4, 120). Engagement of CD24 with Siglec-10 promotes phosphorylation of Siglec-10 ITIM motifs and recruitment of inhibitory molecules including SHP-1 and SH-2, thereby suppressing downstream pathways associated with phagocytic activation, including Syk–PI3K and NF-κB-related signaling, and reducing macrophage-mediated tumor-cell clearance (**Figure 2**) (12). This inhibitory effect depends on both aberrant CD24 expression on tumor cells and Siglec-10 expression in TAMs (12, 121). Importantly, the function of CD24 as a Siglec-10 ligand is not determined solely by protein abundance. Glycosylation and sialylation states can modulate Siglec-10 binding and downstream inhibitory signaling. Thus, both CD24 expression levels and its functional ligand state contribute to the activity of the CD24–Siglec-10 axis.

##### 4.1.1.3 Therapeutic development and translational considerations

Early studies targeting the CD24–Siglec-10 axis provided direct therapeutic proof of concept (12). In co-cultures of MCF-7 breast cancer cells and Siglec-10-positive macrophages, CD24 loss increased tumor-cell susceptibility to phagocytosis, while either anti-Siglec-10 antibodies or SIGLEC10 knockout similarly enhanced macrophage phagocytosis (12). The anti-CD24 monoclonal antibody SN3 also promoted phagocytosis of MCF-7 and other CD24-positive tumor cells, with greater activity than CD47 blockade alone, and combined CD24 and CD47 blockade further increased phagocytosis (12). SN3 likewise enhanced macrophage phagocytosis in mantle cell lymphoma models (122). Attenuation of Fc effector function produced only a modest reduction in phagocytic activity, whereas the F(ab’)_2_ fragment was inactive and direct Siglec-10 targeting had limited effects, indicating that the activity of SN3 cannot be attributed solely to blockade of CD24–Siglec-10 signaling and may also involve Fc-dependent mechanisms (122). These findings are derived primarily from *in vitro* and preclinical models and support partially nonredundant inhibitory inputs from the CD24–Siglec-10 and CD47–SIRPα axes in selected contexts. They do not, however, establish that CD24 blockade is superior to CD47 blockade or that dual-checkpoint inhibition provides clinically meaningful incremental benefit.

###### IMM47

IMM47 is a humanized anti-CD24 monoclonal antibody that has advanced into translational development (84). Preclinical studies showed that IMM47 not only blocks the CD24–Siglec-10 interaction but also retains Fc-dependent effector functions, including ADCC, ADCP, ADCT, and CDC, thereby promoting tumor-cell clearance. Single-agent antitumor activity was observed in xenograft models of colorectal cancer, mantle cell lymphoma, and breast cancer (84). Its *in vivo* antitumor activity was not attributable solely to CD24–Siglec-10 blockade but also depended on effector functions mediated by an intact Fc region (84). Binding of IMM47 to CD24 was relatively insensitive to alterations in N-linked glycosylation within the extracellular domain of CD24 (84). Preclinical studies further reported memory-like antitumor immune responses and enhanced antitumor activity when IMM47 was combined with PD-1 blockade (84). These findings remain preclinical, however, and the clinical benefit of IMM47 has yet to be established. A phase I study of IMM47 in advanced solid tumors (NCT05985083) is currently recruiting.

###### PHST001

Faget et al. (123) recently reported PHST001, a humanized IgG4 anti-CD24 antibody that recognizes CD24 across different glycosylation states and blocks the CD24–Siglec-10 interaction, thereby enhancing macrophage phagocytosis in multiple solid tumor models and patient-derived samples. Unlike the IgG1 antibody IMM47, PHST001 uses an IgG4 format to attenuate Fc effector function; a further Fc-attenuated variant retained pathway-blocking activity but showed reduced pro-phagocytic activity, suggesting that both checkpoint blockade and Fc-related effects contribute to its activity (123). Its efficacy also showed dependence on the macrophage context, with TIM4^+^ tissue-resident macrophages exhibiting higher SIGLEC-G/SIGLEC-10 expression and greater involvement in PHST001-mediated phagocytosis (123). Preclinical studies further showed that PHST001 enhanced the antitumor effects of chemotherapy, radiotherapy, and antibody–drug conjugates (123). PHST001 has entered phase I clinical evaluation in relapsed/refractory solid tumors (NCT06840886), and its clinical benefit and optimal patient population remain to be established.

###### CSBP

Intervention in the CD24–Siglec-10 axis has also been extended to multi-pathway blockade. CSBP (CD24/Siglec-10 blocking peptide) is an optimized D-amino-acid octapeptide that simultaneously interferes with the CD24–Siglec-10 and PD-1–PD-L1 pathways and enhances both myeloid and adaptive antitumor immunity in preclinical models (85). Its antitumor activity exceeded that of anti-PD-L1 or anti-CD24 monotherapy and approached that achieved with their combination (85). The activity of CSBP depended not only on macrophages but also on monocytic myeloid-derived suppressor cells (M-MDSCs), as depletion of MDSCs substantially attenuated its antitumor effect. Combination with radiotherapy further enhanced antitumor activity (85). These findings provide a preclinical mechanistic rationale for further evaluation of CSBP, although its incremental benefit over monotherapy and its efficacy–safety profile remain to be established clinically.

###### AdV-mSS

Local delivery coupled with simultaneous blockade of multiple anti-phagocytic pathways represents another strategy for tumor immunotherapy. The oncolytic adenovirus AdV-mSS enables intratumoral expression and delivery of a bispecific fusion protein that concurrently targets the CD47–SIRPα and CD24–Siglec-10 pathways, enhancing macrophage phagocytosis and antitumor activity in preclinical models of hepatocellular, colorectal, and lung cancers (86). AdV-mSS also promoted reprogramming of TAMs toward an antitumor phenotype, increased MHC-I and MHC-II expression, and enhanced CD8^+^ T-cell infiltration and activation (86). This approach illustrates the combined use of multi-pathway intervention and localized checkpoint blockade, but the supporting evidence remains predominantly preclinical. Whether it can improve the therapeutic window or provide clinical benefit beyond the corresponding single-agent approaches has yet to be established.

The CD24–Siglec-10 axis is among the better-characterized Siglec-dependent glyco-inhibitory checkpoints, and therapeutic strategies targeting this pathway have expanded across multiple translational platforms. Beyond contributing to tumor-cell escape from macrophage phagocytosis, therapeutic modulation of this axis may also influence the broader tumor immune microenvironment. Its clinical translation, however, remains limited by uncertainty regarding pathway dependence and attribution of therapeutic effects. The function of CD24 as a Siglec-10 ligand is determined not only by its expression level but also by its glycosylation and sialylation state (117–119); CD24 expression alone may therefore be insufficient to capture the functional state of the CD24–Siglec-10 interaction. Siglec-10 can also recognize sialylated glycoprotein ligands other than CD24, and the dominant ligand may vary across tumor contexts (17). In addition, anti-CD24 antibodies can promote tumor-cell clearance through Fc-dependent effector mechanisms (84), complicating assessment of the independent contribution of checkpoint relief to therapeutic responses. Future studies should integrate CD24 expression and functional ligand state, the characteristics of Siglec-10-positive myeloid cells, and other Siglec-10 ligands within the tumor context to define pathway dependence and develop appropriate patient-stratification strategies.

#### 4.1.2 α3β1 integrin–Siglec-10 axis

Beyond CD24, recent studies identified α3β1 integrin, composed of ITGA3 and ITGB1, as a functional glycoprotein ligand for Siglec-10 in pancreatic ductal adenocarcinoma (PDAC) (17, 124). In PDAC cells with low or absent CD24 expression, ITGA3 and ITGB1 can still engage Siglec-10 in a sialic acid-dependent manner; ITGA3 knockout or reduced ITGA3/ITGB1 expression decreases Siglec-10 binding and enhances macrophage phagocytosis (**Figure 2****) (**17). These findings support a role for the α3β1 integrin–Siglec-10 axis in phagocytic evasion in PDAC and highlight the tumor-context dependence of Siglec-10 ligands.

In PDAC models, Siglec-10 blockade with 68A11A1 enhances phagocytosis more effectively than anti-CD24 treatment, suggesting that receptor-targeted intervention may block a broader range of inhibitory signals in the presence of multiple ligands (17). However, evidence for this axis remains largely preclinical and confined to PDAC, and its generalizability across tumor types and therapeutic value remain to be established.

#### 4.1.3 GD2–Siglec-7 axis

##### 4.1.3.1 Structure and physiological function

Disialoganglioside GD2 is a glycosphingolipid containing two sialic acid residues and is composed of a ceramide moiety and an oligosaccharide chain (125, 126). Its physiological expression is relatively restricted, occurring primarily in the central nervous system, peripheral sensory nerve fibers, melanocytes, and selected mesenchymal cells (127, 128).

Siglec-7, also known as CD328, is an inhibitory sialic acid-binding receptor expressed predominantly on NK cells, macrophages, and subsets of myeloid dendritic cells (126, 129). Its cytoplasmic region contains canonical ITIMs that, upon recognition of α2,3- or α2,8-linked sialylated glycans, become phosphorylated and recruit downstream inhibitory molecules, thereby restraining immune-cell activation and contributing to immune homeostasis (106, 130, 131). The disialylated structure of GD2 enables it to function as a glycolipid ligand for Siglec-7, providing a molecular basis for glycolipid-dependent immune regulation (130).

##### 4.1.3.2 Functional hijacking in tumors and translational considerations

Recent work has linked the GD2–Siglec-7 axis to macrophage-mediated tumor immune evasion. In osteosarcoma models, tumor-cell-surface GD2 engaged Siglec-7 on macrophages and was associated with increased SHP-2 phosphorylation/activation, reduced macrophage phagocytosis, and enhanced pulmonary metastasis (**Figure 2****) (**132). Reducing GD2 levels or interfering with Siglec-related signaling increased the susceptibility of osteosarcoma cells to macrophage phagocytosis (132). Unlike glycoprotein Siglec ligands such as CD24 and α3β1 integrin, GD2 is a glycolipid, indicating that distinct classes of tumor-associated sialylated surface molecules can provide inhibitory inputs to Siglec receptors (17, 114) and broadening the molecular basis of Siglec-dependent glyco-inhibitory checkpoints. 

Direct evidence for GD2–Siglec-7 as an anti-phagocytic pathway currently derives mainly from preclinical osteosarcoma studies and is insufficient to establish a similar functional role in other GD2-positive tumors. The GD2–Siglec-7 axis should therefore be regarded as an emerging candidate phagocytosis checkpoint whose tumor applicability and therapeutic relevance remain to be defined.

## 5 Glycocalyx-mediated physical barriers

### 5.1 CD43-mediated glyco-immune barrier

#### 5.1.1 Structural basis of the glycocalyx barrier

CD43 is a highly glycosylated transmembrane sialomucin expressed predominantly on leukocytes and their hematopoietic progenitors (165, 166). Its extracellular domain is rich in serine and threonine residues and contains numerous potential O-glycosylation sites, generating a highly glycosylated and sialylated cell-surface structure (167, 168). These features confer a highly extended, negatively charged extracellular architecture that can influence cell–cell contact and receptor–ligand interactions.

The immunological functions of CD43 are strongly dependent on cellular and environmental context, and CD43 is not a canonical inhibitory receptor (13, 169). In the regulation of phagocytosis, its activity derives primarily from its highly extended and sialylated extracellular structure rather than from intracellular inhibitory signaling transmitted to macrophages through a canonical inhibitory receptor–ligand interaction. CD43-mediated resistance to phagocytosis is therefore better conceptualized as a noncanonical glycocalyx barrier (**Figure 2**).

#### 5.1.2 Evidence in tumors and translational considerations

Direct evidence for CD43-mediated resistance to phagocytosis currently derives primarily from AML models. Using genome-wide CRISPR screens, Chung et al. (13) identified O-linked glycosylation and terminal sialylation as major negative regulators of antibody-independent cellular phagocytosis (AICP) and ADCP, with SPN, which encodes CD43, emerging as a key effector gene. CD43 knockout or disruption of key O-glycosylation genes, including C1GALT1 and C1GALT1C1, enhanced macrophage phagocytosis of AML cells and prolonged survival in transplantation models (13). Unlike canonical receptor–ligand phagocytosis checkpoints, CD43 acts primarily through its highly extended, sialylated, and negatively charged extracellular glycocalyx, which limits macrophage–tumor cell contact and phagocytic synapse formation through steric and electrostatic effects and does not depend on SIGLEC-1, SIGLEC-7, or SIGLEC-9 in the experimental systems examined (13). Anti-CD43 antibodies also enhanced macrophage phagocytosis of AML cells, with part of this effect retained after attenuation of FcγR-dependent opsonization, suggesting that interference with the CD43-associated glycocalyx barrier may increase susceptibility to phagocytosis; the precise mechanism, however, has not been directly established (13). The CD43-associated glycocalyx can also restrict NK- and T-cell cytotoxicity against leukemia cells (13).

Current evidence therefore supports CD43 as a noncanonical glycocalyx-mediated anti-phagocytic barrier in AML, but remains largely confined to preclinical AML models, and its generalizability to other tumor types is unclear. Because CD43 and related glycosylation programs are also present in normal hematopoietic cells (165, 166), therapeutic targeting of this mechanism may be constrained by tumor selectivity and the therapeutic window. CD43 is therefore better regarded at present as an emerging candidate anti-phagocytic mechanism rather than a broadly validated phagocytosis checkpoint.

## 6 Shared mechanisms and translational challenges

Inhibitory phagocytosis checkpoints differ in molecular architecture and proximal signaling mechanisms. Receptors including SIRPα, SLAMF3/4, and Siglec-7/10 can recruit SHP-1 and/or SHP-2 to transmit inhibitory signals that attenuate downstream programs associated with phagocytic activation (15, 17, 132, 170–172). CD200R1 instead limits pro-phagocytic receptor signaling primarily through the NPXY–Dok pathway and downstream negative regulatory modules (14), whereas CD43 acts mainly through a highly extended and sialylated extracellular glycocalyx that creates steric and electrostatic barriers (13). LILRB1 and PD-1 can likewise suppress macrophage phagocytosis or related effector functions (16, 135), but current evidence is insufficient to establish direct convergence on SHP-1 and SHP-2 signaling in the relevant phagocytosis models. Despite these differences in proximal mechanism, these pathways can ultimately restrict phagocytosis at the levels of pro-phagocytic signaling, cell–cell contact, cytoskeletal remodeling, or phagocytic synapse formation, collectively forming an anti-phagocytic network within tumors (**Figure 2**).

This network architecture also helps explain why blockade of a single phagocytosis checkpoint does not necessarily produce sufficient or durable antitumor activity. Macrophage phagocytosis is determined by the net balance between inhibitory inputs and pro-phagocytic signals (1, 2). Even when one “don’t eat me” signal is relieved, low tumor-antigen density, insufficient FcγR-mediated pro-phagocytic signaling, or impaired macrophage function may still prevent effective phagocytosis. Multiple anti-phagocytic pathways can also coexist within the same tumor microenvironment, allowing inhibitory inputs to persist after blockade of a single pathway (91). Experience with CD47-targeted therapy further illustrates this translational complexity, including the antigen sink and on-target/off-tumor effects arising from broad expression in normal tissues, RBC clearance, trade-offs between Fc effector function and safety, and the lack of reliable biomarkers of pathway dependence (5, 68, 90, 91). Enhanced phagocytosis in preclinical models or tumor-response signals in early nonrandomized studies should not be regarded as surrogates for durable clinical benefit.

Macrophage heterogeneity and spatial organization further complicate dependence on phagocytosis checkpoints. Single-cell and spatial omics studies have identified distinct transcriptional and metabolic states of TAMs, including C1QC^+^ and SPP1^+^ populations (173, 174), which occupy different spatial niches within tumors (175). Further analyses showed that, within SPP1^+^ TAMs, SIRPα^+^ subsets exhibited greater enrichment of phagocytosis-related and immunosuppressive transcriptional programs and closer spatial proximity to tumor cells (176). These observations suggest that TAM functional state and the spatial organization of receptor–ligand interactions may jointly influence the activity of anti-phagocytic pathways such as CD47–SIRPα. Different TAM states may also exhibit distinct patterns of inhibitory-receptor and Fc-receptor expression and phagocytosis-related programs. However, systematic evidence linking specific TAM subsets to stable dependence on individual phagocytosis checkpoints remains lacking, and whether particular TAM states exhibit preferential checkpoint dependence requires further validation.

This context dependence is particularly evident for Siglec-dependent glyco-inhibitory checkpoints. Siglec pathway activity is determined not only by ligand expression but also by glycosylation and sialylation states and the availability of alternative ligands (17, 113). For example, CD24 and α3β1 integrin can provide inhibitory inputs to Siglec-10 in different tumor contexts (**Figure 2****) (**17). Expression of a single ligand may therefore be insufficient to capture the functional state of the pathway. Future patient stratification may need to integrate tumor ligands and their functional states, the corresponding receptor-positive TAM subsets, pro-phagocytic signaling, the FcγR context, and spatial receptor–ligand proximity rather than relying on expression of a single target.

These complexities indicate that combination strategies should be grounded in demonstrable pathway co-dependence and mechanistic complementarity rather than treating blockade of additional checkpoints as a therapeutic objective in itself. Combination intervention has a clear mechanistic rationale when distinct inhibitory pathways provide demonstrable parallel inputs within the same tumor, or when checkpoint relief requires additional FcγR-mediated pro-phagocytic drive from a tumor-targeting antibody. However, simultaneous disruption of multiple inhibitory pathways may also compromise immune protection of normal tissues. Concurrent loss of SLAMF receptors and CD47 further enhanced phagocytosis but was accompanied by more pronounced depletion of hematopoietic cells (15), illustrating that greater phagocytic activity does not necessarily translate into a broader therapeutic window. Evaluation of combination strategies should therefore shift from asking whether they produce stronger preclinical phagocytic effects to determining whether they provide verifiable incremental clinical benefit in patients with demonstrable pathway dependence while maintaining acceptable normal-tissue safety.

## 7 Conclusions and perspective

Research on inhibitory phagocytosis checkpoints has expanded from canonical receptor–ligand “don’t eat me” signals to Siglec-dependent glyco-inhibitory checkpoints and a noncanonical glycocalyx barrier (12–14, 101), demonstrating that tumor resistance to phagocytosis is not governed by a single pathway but is strongly mechanism- and context-dependent. The maturity of evidence remains highly uneven across targets. CD47–SIRPα has accumulated the most extensive clinical translational experience (6, 8, 10, 11), whereas most emerging mechanisms remain at preclinical or early stages of development (13–15).

Future progress will depend less on identifying additional blockable checkpoints than on defining which anti-phagocytic mechanisms individual tumors functionally depend on while enhancing tumor-cell clearance without compromising immune protection of normal tissues. Achieving this goal will require patient stratification that integrates tumor ligands and their functional states, TAM heterogeneity and spatial organization, pro-phagocytic signaling, and the FcγR context. Therapeutic selectivity may be further improved through optimization of epitopes and binding configurations, Fc and valency engineering, bispecific designs, and tumor-directed or localized delivery. The clinical value of multi-checkpoint and other combination strategies remains to be established through mechanism-guided patient selection and comparative clinical studies.
